# A Smart Archive Box for Museum Artifact Monitoring Using Battery-Less Temperature and Humidity Sensing

**DOI:** 10.3390/s21144903

**Published:** 2021-07-19

**Authors:** Dinesh R. Gawade, Steffen Ziemann, Sanjeev Kumar, Daniela Iacopino, Marco Belcastro, Davide Alfieri, Katharina Schuhmann, Manfred Anders, Melusine Pigeon, John Barton, Brendan O’Flynn, John L. Buckley

**Affiliations:** 1 Tyndall National Institute, University College Cork, Cork T12 R5CP, Ireland; sanjeev.kumar@tyndall.ie (S.K.); daniela.iacopino@tyndall.ie (D.I.); marco.belcastro@tyndall.ie (M.B.); davide.alfieri@tyndall.ie (D.A.); melusine.pigeon@tyndall.ie (M.P.); john.barton@tyndall.ie (J.B.); brendan.oflynn@tyndall.ie (B.O.); john.buckley@tyndall.ie (J.L.B.); 2 ZFB Zentrum für Bucherhaltung GmbH, Bücherstraße 1, 04347 Leipzig, Germany; ziemann@zfb.com (S.Z.); k.schuhmann@zfb.com (K.S.); anders@zfb.com (M.A.)

**Keywords:** battery-less NFC sensor, cultural heritage objects, energy harvesting, high-frequency RFID, museum artifacts monitoring, preventive conservation, sensor technology, smart archive box

## Abstract

For the first time, this paper reports a smart museum archive box that features a fully integrated wireless powered temperature and humidity sensor. The smart archive box has been specifically developed for microclimate environmental monitoring of stored museum artifacts in cultural heritage applications. The developed sensor does not require a battery and is wirelessly powered using Near Field Communications (NFC). The proposed solution enables a convenient means for wireless sensing with the operator by simply placing a standard smartphone in close proximity to the cardboard archive box. Wireless sensing capability has the advantage of enabling long-term environmental monitoring of the contents of the archive box without having to move and open the box for reading or battery replacement. This contributes to a sustainable preventive conservation strategy and avoids the risk of exposing the contents to the external environment, which may result in degradation of the stored artifacts. In this work, a low-cost and fully integrated NFC sensor has been successfully developed and demonstrated. The developed sensor is capable of wirelessly measuring temperature and relative humidity with a mean error of 0.37 °C and ±0.35%, respectively. The design has also been optimized for low power operation with a measured peak DC power consumption of 900 μW while yielding a 4.5 cm wireless communication range. The power consumption of the NFC sensor is one of the lowest found in the literature. To the author’s knowledge, the NFC sensor proposed in this paper is the first reporting of a smart archive box that is wirelessly powered and uniquely integrated within a cardboard archive box.

## 1. Introduction

The degradation rate of movable tangible Cultural Heritage (CH) objects and artifacts can considerably increase due to exposure to unstable climatic conditions, light and environmental pollutants [1]. As a well-known example, acidic historic papers and documents containing iron gall ink are prone to deterioration when exposed to temperature and humidity fluctuations [2,3]. Variations of ±10% in Relative Humidity (RH) and ±5 °C in temperature at RH values below 65% are generally considered acceptable for paper based artifacts [4]. However, the majority of precious documents and artifacts owned by museums and archives are often stored in climatically uncontrolled storage areas and in museum building basements for example. The implementation of modern air conditioning systems and Heating, Ventilation, and Air Conditioning (HVAC) systems constitutes a highly technical conservation challenge in itself that often cannot be financially justified, especially for small and medium-size institutions. This paper focuses on the development of a novel environmental sensor (NFC sensor) for microenvironment monitoring and its integration into a smart archive box. 

Monitoring microenvironments is an important tool for preventive conservation, even in climatically controlled areas. However, the microclimatic environmental conditions (such as temperature and humidity) for artifacts stored in archive boxes can differ significantly from that of the external environment due to the intrinsic water content of stored materials within the archive box. A common and legitimate concern of conservators, therefore, is the (often-unknown) microclimate within the enclosures themselves that are used to house valuable artifacts. Consequently, there is a need to monitor the effective interior microclimates in sealed storage enclosures. The measurement of these local temperature and humidity values in real time provides museum owners the opportunity to act quickly and prevent degradation of valuable artifacts due to shrinkage or mold growth for example. Commercially available hygrometers or data loggers and other environmental monitoring devices are well known for monitoring microenvironments in museums during transport or inside storage or display rooms [1,5]. However, these commercially available solutions are not suitable for integration into a large number of storage enclosures because they are either too large or too expensive [6]. Additionally, the storage box needs to be physically opened to enable the reading of sensor data and any required maintenance, such as the changing of batteries. This in itself may cause artifact degradation by introducing contaminants when opening the box. To date, there are no convenient cost-effective battery-less wireless solutions available to solve the above problem in this application domain. 

In recent years, the market for NFC technology has increased significantly due to the incorporation of NFC reader capability within smartphones and the increasing significance of the Internet of Things (IoT) based applications [7]. At present, NFC technology not only enables consumers to perform payments for example, but also helps to passively measure and wirelessly transmit data sets of various parameters such as temperature and humidity for a wide range of IoT application [8,9]. NFC technology has evolved from traditional radio frequency identification (RFID) technologies, and uses inductive coupling to enable data transfer between two NFC loop antennas located in each other’s proximity or vicinity [10]. In particular, High Frequency (HF) NFC technology is a short-range and contactless communication technology, which operates within the worldwide available unlicensed radio frequency band of 13.56 MHz [10]. In passive communication mode, NFC technology offers a data rate of between 106 kbps to 848 kbps with a communication range of less than 10 cm using the ISO14443 standard air interface. NFC also offers a lower data rate of between 6 kbps to 53 kbps using the ISO15693 standard air interface within a range of less than 20 cm [9,11,12,13,14]. The additional NFC standard (ISO/IEC 18092 and ECMA-340) supports data frame formats such as NFC Data Exchange Format (NDEF), modulation, interoperability, and data exchange between standards-based NFC-enabled devices [10,15,16,17,18]. In addition, the NFC forum has developed technical specifications to ensure interoperability between NFC devices and Radio Frequency (RF) test mechanisms [19]. NFC technology is also backward compatible with proximity and vicinity smartcard standards, such as ISO/IEC 14443A, ISO/IEC 14443B, and ISO/IEC 15693 [10]. A benefit of backward compatibility is that an NFC radio transceiver based on the ISO/IEC 15693 standard can transmit data up to 1 m with a 13.56 MHz reader and up to 7 cm with an NFC type 5 enabled smartphone [20]. 

In recent years, various battery-less and battery-assisted wireless sensing solutions have been developed using NFC and RFID technology in multiple application domains. These applications require the sensing of multiple disparate parameters including temperature, humidity, pH, CO_2_, and pressure [21,22,23,24,25,26,27,28,29,30,31,32,33,34,35,36,37,38,39]. Such applications include soil moisture monitoring [40,41,42], fruit quality measurement [43,44], long-term building structural health monitoring [45,46], gas monitoring (ammonia, CO_2_ and oxygen) [47], and environmental parameter monitoring [48,49]. 

In terms of museum artifact monitoring applications [50], the long-term preventive care of fine art objects using an NFC sensor has been reported. In [50], the differences between the temperature and humidity levels inside and outside of the microclimatic storage enclosure have been evaluated using a battery-assisted NFC sensor. Similarly, the identification of cultural relics in museums and the capability for environmental monitoring, using RFID technology, have been reported in [51,52]. However, these reported artifact-monitoring solutions require a battery as a source of power.

A comprehensive review of NFC technology and NFC and HF RFID based commercial NFC sensors for museum artifacts monitoring has recently been reported by the authors in [53]. This paper highlighted several challenges with commercial NFC sensor solutions which have a limited operational life of 3 years maximum [54]. In addition, only a limited number of the reported solutions support battery-less operation [55,56] and are not optimized for low-cost applications [54,55,56,57,58]. The DC power consumption of battery-less NFC sensors reported in the literature is generally higher than 1.5 mW [28,29,30,34,41,42,43,47,53,59,60,61,62,63,64,65]. In [24,38], an NFC sensor power consumption of less than 1 mW has been reported, however the authors did not report the precise value of the power consumption. In museum artifact monitoring, NFC sensor cost, wireless communications range and operational life are all important requirements. In addition, NFC sensor integration in packaging material without using adhesive are vital requirements. The above discussed NFC sensors and commercially available solutions do not meet all of these requirements for the proposed museum artifact monitoring application and therefore a custom solution is required. 

**Figure 1:**
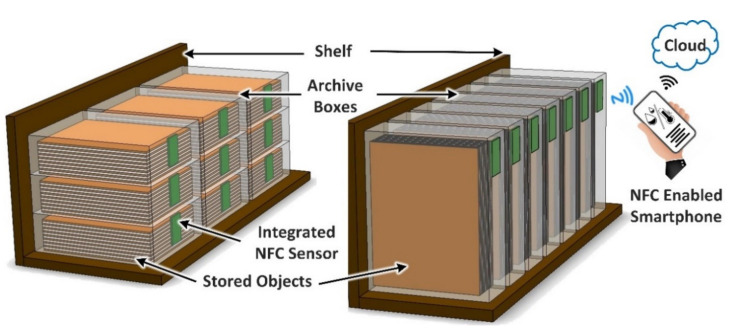
Illustration of the proposed solution for artifact storage monitoring using a novel battery-less temperature and humidity NFC sensor powered using a standard smartphone.

This paper presents a novel smart archive box for museum artifact monitoring of historical, valuable, and paper-based cultural heritage objects. The developed solution implements a battery-less NFC sensor for temperature and humidity monitoring. The sensor has a DC power consumption of 900 μW. Figure 1 illustrates the proposed solution for artifact storage monitoring using a novel battery-less temperature and humidity NFC sensor powered using a standard smartphone. The NFC sensor is integrated directly into the archive box itself. In addition, the smartphone can be used to transfer the measured data to the cloud for further analysis and processing by using a smartphone application. This procedure will enable conservators to monitor microenvironments within the enclosure to help protect the contents. For example, monitoring at regular intervals via the presented approach makes it possible to identify increased interior humidity values at an early stage. This can facilitate the timely de-humidification of the enclosure or even the simple movement to an alternative location in the storage area with better air circulation. 

The proposed smart archive box results in advantages such as low maintenance, low-cost, long-term, and intelligent sensing solution for artifact preservation, storage, and monitoring. 

This paper is organized as follows: Section 2 describes the proposed solution development methods, which include battery-less NFC sensor design and integration into archive boxes. The results of NFC sensor hardware prototype testing are discussed in Section 3. Finally, Section 4 concludes the paper and summarizes the key findings of this work.

## 2. Materials and Methods

This section summarizes the architecture of the proposed NFC sensor system, driven by the user requirements for the application. The system design was tailored for a low-cost implementation using commercial-off-the-shelf (COTS) sensor hardware integrated in a cardboard archive box. The developed NFC sensor hardware prototype comprises the following COTS components: An NFC radio transceiver (ST25DV16K-JFR6D3) [66], microcontroller (STM32L031K6U6) [67], voltage regulator (STLQ015M18R) [68] that are manufactured by STMicroelectronics, Geneva, Switzerland and temperature and humidity sensor (SHTC3) [69] that is manufactured by Sensirion AG, Stäfa, Switzerland. The sensor hardware uses standard, four-layer FR4 substrate printed circuit board (PCB) that was fabricated by ECS Circuits, Dublin, Ireland. More detailed information regarding component selection and cost estimation using the references [8,20,53,66,67,69,70,71,72,73,74,75,76,77,78,79,80] is presented and discussed in detail in the Supplementary Material submitted with this paper.

## 2.1. NFC Sensor User Requirements

The user requirements for the proposed NFC sensor are summarized in Table 1 and are derived from the APACHE project user requirements listed in [81]. The NFC sensor needs a cost of less than EUR 5 per 10k quantities with an operational life of greater than 5 years. An integrated memory (EEPROM) is essential to temporarily store measured temperature, humidity and metadata (such as archive box identification ID, artifact type, and location). The NFC sensor is required to transmit measured data at a minimum data rate of 25 kbps. The presented design does not necessitate a battery, is wirelessly powered by an NFC enabled smartphone, and is required to provide a harvested DC power of greater than 1 mW [53]. 

This enables the smartphone to monitor the internal environment of the archive box by placing the smartphone close to the outside surface of the box. Furthermore, in most small and medium-size museum archive boxes, the available real-estate is limited. Hence, a low-profile ‘credit-card’ realization with dimensions of 85.60 mm × 53.90 mm × 2 mm was used.

## 2.2. Block Diagram of Proposed NFC Sensor

**Figure 2:**
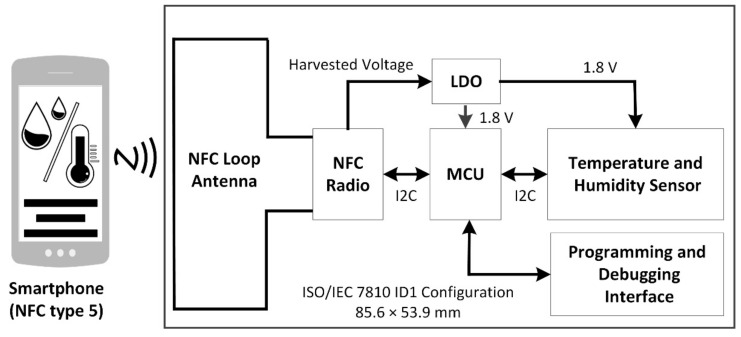
Block diagram of proposed NFC sensor that is powered using a smartphone.

Figure 2 shows a block diagram of the proposed NFC sensor with a NFC enabled smartphone used to wirelessly power the sensor. In operation with the NFC enabled, the smartphone provides wireless power to the NFC loop antenna via inductive coupling. The antenna then provides RF power to the NFC radio. Energy harvesting circuitry within the NFC radio provides a harvested DC output that is then regulated using a low dropout voltage regulator (LDO) that provides a 1.8 V DC output to power the microcontroller, sensor, and all other circuitry. Digital communications between the NFC radio transceiver, microcontroller (MCU) and temperature and humidity sensor are implemented by using the Inter-Integrated Circuit protocol (I2C) [66]. Programming and debugging interfaces are also provided for firmware upload and debugging. Upon power-up of the sensor, temperature and humidity parameters are measured and along with metadata, they are read by the MCU and written to the NFC radio’s integrated non-volatile memory (EEPROM). The measured sensor data can then be read upon command from the smartphone for later transfer display and analysis on the smartphone or later processing in the cloud. 

## 2.3. Low Power Hardware Design Method

This subsection describes the methods used to minimize the DC power consumption of the proposed NFC sensor. It is known [82,83,84,85] that magnetic field strength decays proportionally to the cube of the distance between two mutually-coupled coils (Smartphone Coil and NFC sensor coil). In other words, there is a limit to the available harvested RF power for the NFC sensor for a given distance from the Smartphone. The DC power consumption of the NFC sensor is therefore of key interest and in this work, NFC sensor has been optimized for low power operation as described below. The electrical characteristics of the MCU, NFC radio transceiver, temperature, and humidity sensor reported in the datasheet are summarized in Table 2. From Table 2, it can be observed that the overall DC power consumption for the main components is 11.07 mW using datasheet values for power consumption [66,67,69]. In this case, the microcontroller has an estimated DC power consumption of 8.1 mW, which is 73.17% of the overall DC power consumption for the NFC sensor and therefore needs to be optimized. 

The DC power consumption of digital complementary metal-oxide-semiconductor (CMOS) integrated circuits such as MCUs varies with the square of the supply voltage [86,87]. In addition, the DC power consumption varies in direct proportion with the MCU clock frequency. For this NFC sensor design, both voltage and frequency scaling techniques have been used to minimize DC power consumption with detailed measurement-based information presented in Section 3.2. 

## 2.4. Hardware Prototype of Proposed NFC Sensor

**Figure 3:**
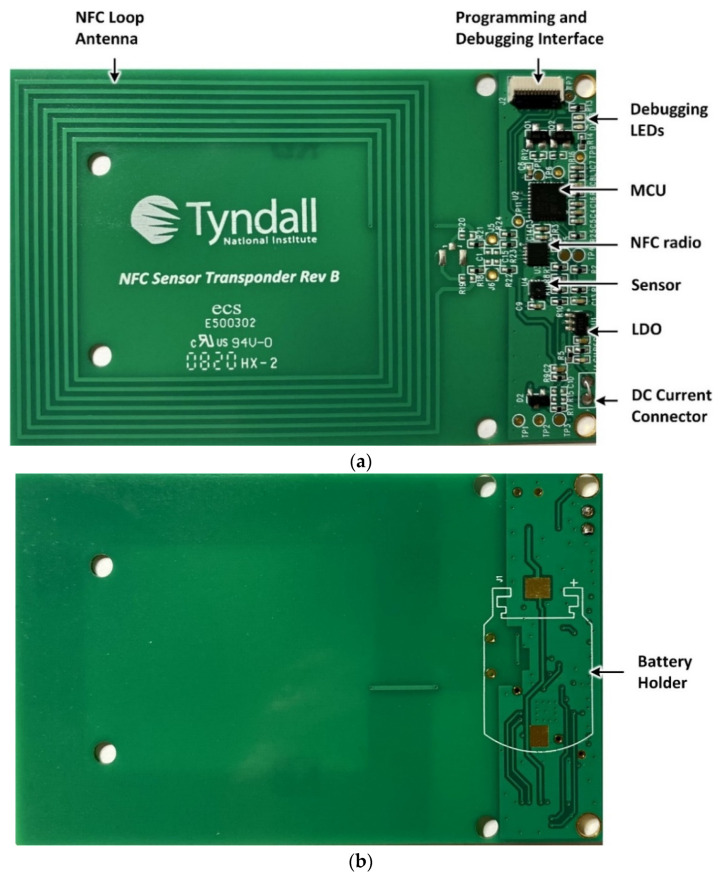
NFC sensor hardware prototype (**a**) Top side and (**b**) Bottom side.

Figure 3 show the hardware prototype of the developed solution, which is implemented in a PCB configuration with dimensions of 85.60 mm × 53.90 mm. The NFC loop antenna is shown on the left of Figure 3a. The inductive loop antenna is designed to have 7 turns, a trace width of *T*_W_ = 0.6 mm, a turn spacing of *T*_S_ = 0.75 mm and a copper thickness of 35 μm that yields a measured inductance of 6.5 μH at 13.56 MHz. An integrated capacitance within the NFC radio transceiver connects in shunt with the loop antenna to form a parallel LC circuit with a nominal resonant frequency of 13.56 MHz. The MCU, sensor (temperature and humidity) and LDO are also shown in addition to the programming interface. A 0.8 mm 4-layer PCB stack-up was employed for this design with all components placed on the top layer to yield a planar structure for ease of later integration within the cardboard archive box. The developed NFC sensor also has the capability for continuous monitoring and data logging of temperature and relative humidity data when an optional coin-cell battery is included as shown in Figure 3b. In this work, the coin-cell battery is not included and data-logging functionality will be characterized in future work.

**Figure 4:**
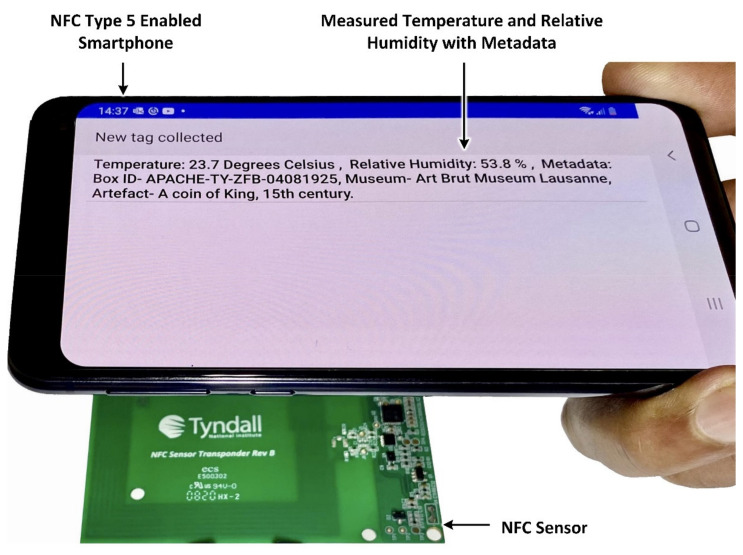
Demonstration of wirelessly powered (battery-less) NFC sensor using smartphone.

Figure 4 demonstrates the prototype NFC sensor being wirelessly powered and read using a smartphone. The smartphone, when placed within a distance of <4.5 cm to the sensor, enables the temperature and relative humidity values and additional metadata to be measured wirelessly, with no requirement for battery power on the sensor. Any smartphone with NFC type 5 capability can operate the sensor as shown.

## 2.5. Integration of the NFC Sensor within a Cardboard Archive Box

The primary motivation for integrating the NFC sensor in museum archive enclosures is to quantify and measure the temperature and relative humidity conditions inside the archive box itself, without having to open the box and disturb the environment. For the successful integration of NFC sensors into archive boxes, the following requirements need to be fulfilled:

1. The NFC sensor needs to be directly exposed to the ‘packed air’ in contact with the stored museum artifact within the box.
2. The measurement of temperature and relative humidity needs to take place at a position that ensures the most representative environmental data within the box.
3. The NFC sensor is required to be readily accessible for wireless reading using the smartphone, even when the boxes are stacked upon each other or lined up together.
4. The NFC sensor is required to be wirelessly connectable even through multiple layers of cardboard box material.
5. The NFC sensor needs to be protected from direct contact with the packed goods within the box to prolong its useful life.
6. The NFC sensor needs to be integrated without the use of adhesives to avoid contamination of the atmosphere inside the box.

The NFC sensor system described in this work focuses on a commonly used standard ‘telescope box’ [88] with an interior size of 10.5 × 25.4 × 33.7 cm (H × W × L) and the following descriptions illustrate the methodology to meet the NFC sensor integration requirements.

**Figure 5:**
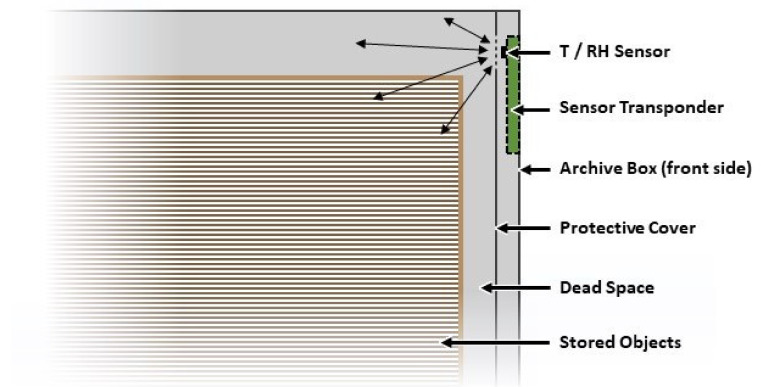
Schematic illustration of a packed archive box with integrated NFC sensor, which is exposed to a maximum of the interior atmosphere surrounding the artifact (indicated by arrows).

Although the storage of objects within archive boxes usually aims to maximize the utilization of the available space, this often leaves little residual volume of atmosphere around the artifact due to the use of standard sized boxes that are manufactured in large quantities. It is therefore advantageous to expose the sensor to residual or ‘dead’ space environment that is directly interacting with the stored artifact. In addition, the positioning of an NFC sensor on the interior of the box needs to provide a convenient means for an operator to read the sensor using a smartphone, without having to move the boxes when they are laid horizontally on the top of the other or stored vertically alongside one another, as depicted in Figure 1. The boxes’ front side, which directly faces the operator, is therefore chosen for the installation of the NFC sensor, since it provides a convenient means to read the NFC sensor by simply placing the smartphone close to the surface of the archive box. Protection of the sensor from direct contact with the packed object (and vice-versa) can be provided by incorporating an additional cover layer of material, as illustrated in Figure 5. 

**Figure 6:**
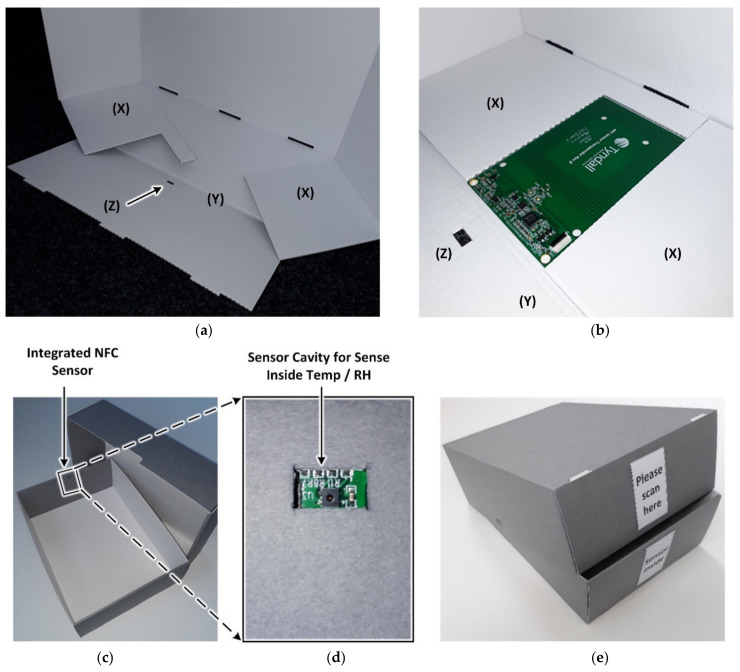
NFC sensor integration (**a**) Short front side of the flipped open telescope box bottom without the NFC sensor, (**b**) with integrated NFC sensor (**c**) integrated view of NFC sensor (**d**) view of sensor cavity for sensing inside temp/RH, and (**e**) labelled base and lid displaying construction assembly for reading.

The NFC sensor is placed in a shape-specific recess in one of the fastening flaps ‘(X)’, as shown in Figure 6a,b, which are folded inside to be covered by the front flap ‘(Y)’. This location represents the operator-facing front side of the box and the sensor faces towards the interior space to sample the environmental conditions within the box. An aperture on flap ‘(Z)’ is indicated by the arrow in Figure 6 used to position the temperature and humidity sensor in air contact within the box. For any particular NFC sensor size and sensor location, the recess in flap ‘(X)’ and the position of cavity ‘(Z)’ can be customized. The integration of the upright NFC sensor shown best fulfils the requirement number 2 but is, however, limited to telescope boxes possessing a height of 9 cm and flap boxes possessing a width of 5 cm minimum, according to the NFC sensor size. A label indicating the location of the NFC sensor is used to guide the operator to position the smartphone during a NFC sensor read operation. 

The sensor integration described permits a reliable detection of microclimates within the archive box to which the artifact is exposed. While its unhindered exposure to the enclosed atmosphere is ensured, the direct physical contact between the sensor and the stored object is avoided. This not only protects the artifact from the electronics, but also protects sensitive electronic components and circuits. A straight-forward and simple construction of the collapsible enclosure is presented, which is comparable to conventional archive boxes which are well known to conservators. It does not require any additional components or adhesives and can be offered as a low cost uncomplicated modular system of ‘box + sensor’. With this interdisciplinary approach, the proposed battery-less archive box with integrated NFC sensor enables a low-cost, long term and intelligent solution for artifact storage and monitoring.

## 3. Results and Discussion

This section presents the measured performance of the developed prototype NFC sensor. The testing was used to test the accuracy of temperature and relative humidity sensor measurements against a calibrated standard. Power consumption and wireless communication range performance measurement results for the NFC sensor are also presented.

### 3.1. Temperature and Relative Humidity Measurement Using the Prototype NFC Sensor

**Figure 7:**
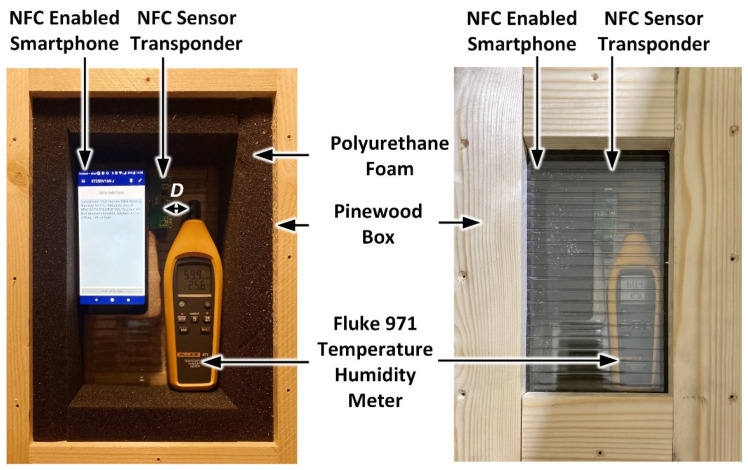
NFC sensor temperature and relative humidity measurement setup.

In order to determine the accuracy of the developed prototype sensor, the measured temperature and relative humidity values have been compared with a calibrated Fluke 971 temperature and humidity meter [89]. The testing was performed in the setup shown in Figure 7 using an insulated pinewood enclosure [90,91]. The NFC and 971 meter sensors were placed in close proximity within a distance D of 2.5 cm.

**Figure 8:**
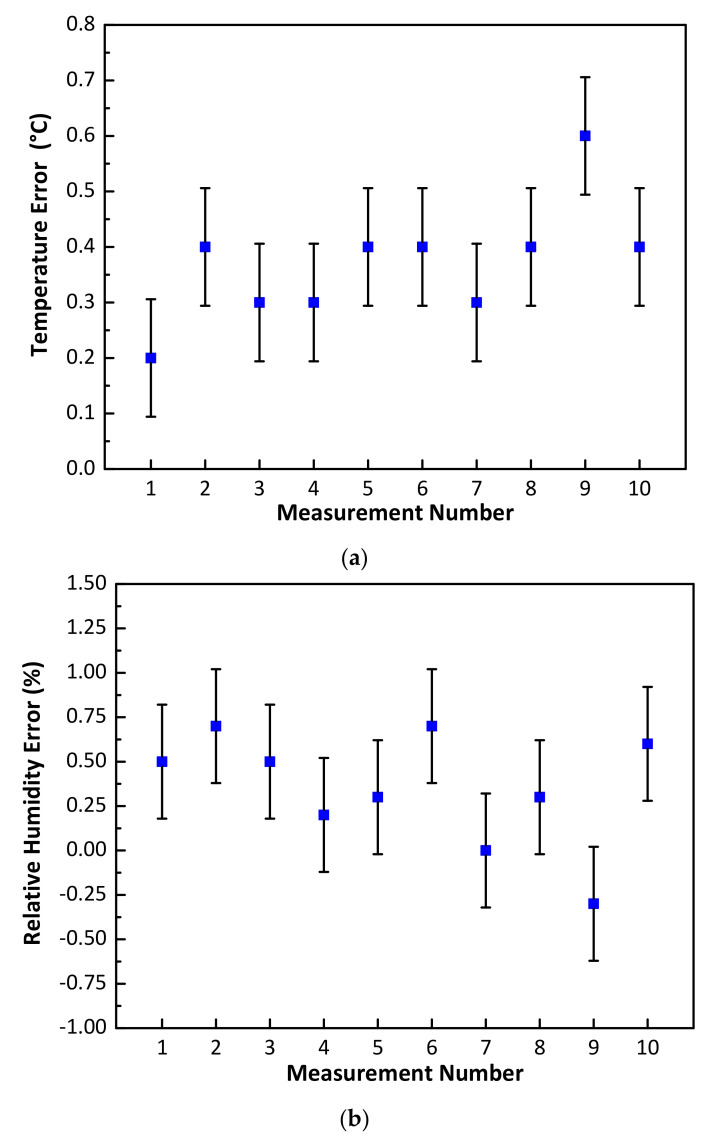
NFC sensor measurement comparison with a calibrated Fluke 971 meter (**a**) temperature error (°C) and (**b**) Relative humidity error (%).

Initially, the NFC sensor, along with the smartphone (Vodafone X9) and Fluke 971 meter were placed within the enclosure for a period of 60 minutes. During NFC sensor measurements, the transparent lid of the pinewood box was removed and the smartphone was placed over the NFC sensor to capture the measured temperature and relative humidity of the NFC sensor within a period of less than one minute. The measured temperature and relative humidity for the Fluke 971 meter were also recorded and the lid was subsequently closed. Using this approach, 10 measurements for temperature and relative humidity were taken over a one-hour period. During measurements, the ambient lab temperature and relative humidity varied between 24.6 °C to 26.5 °C and 59.9% to 63.8%, respectively. The measured temperature and relative humidity errors between the NFC and calibrated meter measurements are shown in Figure 8. Figure 8a shows that a minimum error of 0.2 °C and a maximum error of 0.6 °C was recorded. Similarly, the measured relative humidity results are shown in Figure 8b with a minimum error of 0% and maximum error of 0.7% being recorded. The above results show that the NFC sensor exhibited a mean error of 0.37 °C and a standard deviation of 0.106 °C in temperature and a mean error of ± 0.35% and a standard deviation of 0.321% in relative humidity. In Figure 8, the vertical bar symbols (in black) represent the magnitude of standard deviation and the blue square symbols denote the mean error value.

The temperature accuracy of the developed NFC sensor was also characterized from 6.8 to 50.6 °C as outlined in detail in Section S3 of the Supplementary Material submitted with this paper. The results of this experimental work show a measured mean error in temperature of ±0.34 °C from 6.8 to 50.6 °C. The humidity sensor (SHTC3) used in the NFC prototype design covers a relative humidity range of 20 to 80% with a typical accuracy of ±2% at temperatures 25 °C [69]. Figure S3 in the Supplementary Material also summarize the typical accuracy of relative humidity measurements for temperatures ranging from 0 °C to 80 °C. In addition, Figure S4 summarize the typical accuracy of relative humidity measurements at temperatures 25 °C. Both figures provided from the Sensirion SHTC3 datasheet [69], with the SHTC3 sensor fully calibrated to meet the specification described in Application Note [92]. 

### 3.2. DC Power Consumption and Wireless Communications Range

**Figure 9:**
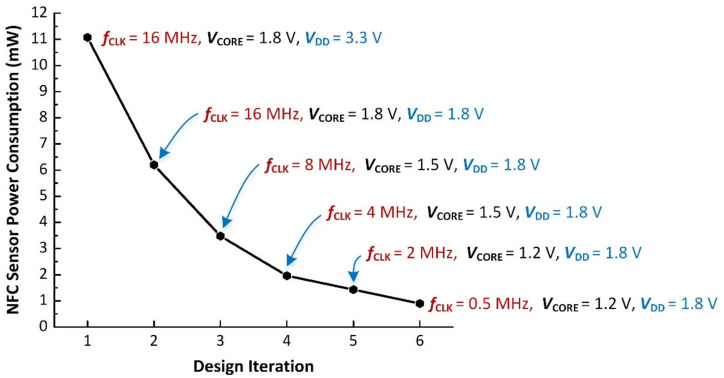
Measured NFC sensor DC power consumption using MCU voltage and frequency scaling.

In order to decrease the DC power consumption of the developed NFC sensor, voltage and frequency scaling techniques have been employed as discussed in Section 2.3. Figure 9 shows the measured DC power consumption as a function of MCU voltage and clock frequency. As shown in Figure 9, DC power consumption of 11.07 mW was observed at the first design iteration. In the second iteration, *V*_DD_ was reduced to 1.8 V (with *V*_CORE_ = 1.8 V and *f*_CLK_ = 16 MHz), reducing the DC power consumption to 6.20 mW. Further, design iterations reduced the clock frequency and core voltage as shown in Figure 9. The lowest DC power consumption was achieved at *f*_CLK_ = 0.5 MHz, *V*_CORE_ = 1.2 V, and *V*_DD_ of 1.8 V. The final optimized NFC sensor has a measured DC power consumption of approximately 900 μW when the smartphone was placed at a distance of approximately 2 cm from the NFC sensor. As shown in Figure 9, the use of the described frequency and voltage scaling techniques enable a factor of 12.3 reduction in DC power consumption for the NFC sensor. 

In order to measure the wireless communications range, a Samsung Galaxy S10 smartphone was used. The maximum wireless communication range obtained for the NFC sensor was measured at 4.5 cm in free-space with the NFC sensor operating at optimal settings for low power, namely *f*_CLK_ = 0.5 MHz, *V*_CORE_ = 1.2 V, and *V*_DD_ of 1.8 V. 

### 3.3. Benefits and Convenience of Smart Archive Box to the User

In terms of the developed NFC sensor, it is also instructive to highlight the advantages of the smart archive box to the user and the convenience afforded as described in this subsection:

1. The proposed temperature and humidity sensing solution is the first reporting of a smart archive box of its type in the literature for museum artifact monitoring.
2. The proposed method uses a battery-less NFC sensor that can be read conveniently with a standard NFC-enabled smartphone.
3. There is no requirement for a battery and the archive box does not need to be opened during the measurement so the internal environment is un-disturbed.
4. Another convenient feature is that there is no requirement for maintenance of the NFC sensor such as battery replacement. This could be problematic in a situation where thousands of stored items existed in a large collection for example, requiring significant maintenance and personnel costs.
5. A low-cost sensor has been developed in this work with a cost of €4.91 in volumes of 10 thousand units making it suitable for small to medium-sized museums.
6. The removal of the requirement for a battery means that there is a positive environmental impact with no need to dispose of depleted batteries.
7. The presented solution using passive wireless sensing is convenient for many small and medium sized institutions, making it possible to measure important artifact environmental data without large costs and effort being required.
8. Several improvements are planned for future work to continue developing in the direction of continuous sensing/monitoring. However, the present solution meets many requirements of many institutions

## 4. Conclusions

For the first time, this paper presents a novel smart archive box for microclimate monitoring in museum artifact storage applications. The developed NFC sensor does not require a battery and is wirelessly powered using a standard smartphone. A low-cost prototype NFC sensor has been successfully developed, demonstrated and tested. The developed NFC sensor is uniquely integrated in a low-cost cardboard archive box for real-world deployments. With a measured mean error of ± 0.34 °C in temperature from 6.8 °C to 50.6 °C, the proposed NFC sensor qualifies for accurate temperature measurements for the intended application. Similarly, the relative humidity accuracy is ± 2% from 20% to 80% relative humidity using datasheet values for the sensor. The developed NFC sensor has also been optimized for low power operation using voltage and frequency-scaling techniques, resulting in a peak DC power consumption of 900 μW, which is one of the lowest found in the literature. Wireless communications performance tests on the prototype sensor show a maximum range of 4.5 cm by using a Samsung Galaxy S10 smartphone. 

The developed low-cost solution is especially beneficial for small and middle-size museums with critical or inappropriate storage climates and budget constraints that preclude the use of expensive air conditioning systems. The proposed solution described in this paper, for the first time, enables accurate microclimate environmental monitoring without the need for movement or opening of the archive box and, in addition, does not require battery replacements. This feature is especially advantageous for museum conservators to readily monitor and reduce the risk of degradation of valuable cultural heritage artifacts in storage.

Future work will fully characterize the temperature and relative humidity accuracy of the developed sensor using an environmental chamber test system, which was not available at the time of writing. In addition, future work will investigate methods for automatic data collection solutions to improve user convenience. The system-level response time for the developed NFC sensor will be studied in future work. For continuous monitoring, firmware updates and smartphone application development are required in future work.

## Figures and Tables

**Table 1:** NFC sensor technical and user requirements.

Requirements	Values
Power Source	Wireless power transfer using NFC
DC Power	>1 mW
NFC sensor cost	<€5 in 10k quantities
NFC sensor operational Life	>5 years
Wireless communication Range	<4 cm
User memory (EEPROM)	<20 kbits RF and I2C dual interface access
Wireless communication data rate	25 kbps
Communication standard	ISO/IEC 15693 and NFC Type-5
NFC sensor form factor	85.60 mm × 53.90 mm × 2 mm
NFC sensor packaging	Encapsulation in cardboard box packaging material without adhesive
Sensed environmental parameters	Temperature and Humidity
Temperature accuracy	<±0.5 °C
Relative humidity accuracy	<±1%
Operational temperature range	–40 to +125 °C [69]
Operational humidity range	0 to 100% [69]
Response time at τ (63%)	8 s for relative humidity, 5 to 30 s for temperature [69]
Sensor parameter reading	NFC wireless using Smartphone

**Table 2:** Electrical specifications for IC components (datasheet) in developed NFC Sensor.

Component and Part Number	Maximum Power Consumption (mW)	Operating Condition	Supply VOLTAGE Range (V)	Datasheet Reference
MCU (STM32L031K6U6)	8.1	*V*_DD_ = 3.0, *V*_CORE =_ 1.8, *f*_CLK =_ 16 MHz	1.65–3.6	[67]
NFC radio transceiver (ST25DV16K-JFR6D3)	1.089	*V_DD_* = 3.3, *f_C_* (I2C) = 1 MHz (<50 ns), Write operation	1.8–5.5	[66]
Sensor (SHTC3)	1.881	Low power mode, *V_DD_* = 3.3	1.62–3.6	[69]

## Supplementary Materials

The following are available online at https://www.mdpi.com/article/10.3390/s21144903/s1, Section S1: Component selection and proof of concept of NFC sensor, Section S2: NFC sensor cost estimation, Table S1. Developed NFC sensor cost estimation at 10k quantities. Section S3: Temperature measurement using the prototype NFC sensor. Figure S1. NFC sensor temperature measurement setup (a) Outside view (b) Inside view. Figure S2. NFC sensor temperature measurement comparison with a calibrated Fluke 971 meter. Section S4: Typical accuracy of relative humidity measurements. Figure S3. Typical accuracy of relative humidity measurements given in % RH for temperatures 0 °C to 80 °C [69]. Figure S4. Typical and maximal tolerance for relative humidity at 25 °C [69].
