# Supplementary material for: A Smart Archive Box for Museum Artifact Monitoring Using Battery-Less Temperature and Humidity Sensing
Source: Sensors (Basel). 2021 Jul 19;21(14):4903. doi: 10.3390/s21144903 (PMC8309753; doi:10.3390/s21144903)
Supplement: Supplementary file 1 [file sensors-21-04903-s001.zip › sensors-1263114-supplementary.pdf]

sensors MDPI

Supplementary Materials:

A Smart Archive Box for Museum Artifact Monitoring using Battery-less NFC Sensing

Dinesh R. Gawade [1*], Steffen Ziemann [2], Sanjeev Kumar [1], Daniela Iacopino [1], Marco Belcastro [1], Davide Alfieri [1], Katharina Schuhmann [2], Manfred Anders [2], Melusine Pigeon [1], John Barton [1], Brendan O'Flynn [1] and John L. Buckley [1]

[1] Tyndall National Institute, University College Cork, Cork, T12 R5CP, Ireland; sanjeev.kumar@tyndall.ie (S.K.); daniela.iacopino@tyndall.ie (D.I.); marco.belcastro@tyndall.ie (M.B.); davide.alfieri@tyndall.ie (D.A.); melusine.pigeon@tyndall.ie (M.P.); john.barton@tyndall.ie (J.B.); brendan.oflynn@tyndall.ie (B.O.); john.buckley@tyndall.ie (J.L.B.)
[2] ZFB Zentrum für Bucherhaltung GmbH, Bücherstraße 1, 04347 Leipzig, Germany; ziemann@zfb.com (S.Z.); k.schuhmann@zfb.com (K.S.); anders@zfb.com (M.A.)
* Correspondence: dinesh.gawade@tyndall.ie; Tel.: +353-21-234-6442

Citation: Gawade, D.R.; Ziemann, S.; Kumar, S.; Iacopino, D.; Belcastro, M.; Alfieri, D.; Schuhmann, K.; Anders, M.; Pigeon, M.; Barton, J.; et al. A Smart Archive Box for Museum Artifact Monitoring using Battery-Less Temperature and Humidity Sensing. *Sensors* **2021**, *21*, 4903. https://doi.org/10.3390/s21144903

Academic Editor: VMN Passaro

Received: 31 May 2021
Accepted: 12 July 2021
Published: 19 July 2021

Publisher's Note: MDPI stays neutral with regard to jurisdictional claims in published maps and institutional affiliations.

Copyright: © 2021 by the authors. Licensee MDPI, Basel, Switzerland. This article is an open access article distributed under the terms and conditions of the Creative Commons Attribution (CC BY) license (http://creativecommons.org/licenses/by/4.0/).

S1: Component selection and proof of concept of NFC sensor

In developing, the NFC sensor architecture to meet the requirements in Table 1, the selection of appropriate commercial-off-the-shelf (COTS) electronic components was critical. To identify a suitable temperature and humidity sensor for this application, the technical specifications of several temperature and humidity sensors were studied and matched against the user requirements as defined Table 1. From the conclusions of this study, the Sensirion SHTC3 temperature and humidity sensor that is manufactured by Sensirion AG, Stäfa, Switzerland [69] has been chosen because of its low DC supply voltage (1.8 V), low DC current consumption (270 μA) and high temperature, and relative humidity accuracy, which is 0.2°C and 2 %, respectively. An added advantage of SHTC3 sensor is its small size (2 × 2 × 0.75 mm). Similarly, the STM32L03 series microcontroller that is manufactured by STMicroelectronics, Geneva, Switzerland was chosen because of its ultra-low power consumption. The STM32L031K6U6 microcontroller has the capability to operate down to 49 μA/MHz using an external DC/DC converter and 76 μA/MHz using low drop voltage regulators in dynamic run mode [67].

In order to select a suitable NFC radio transceiver for the intended application, the technical specifications of various NFC radio transceiver were first evaluated against the user requirements. Further, communication range and power consumption experiments were conducted with potential NFC radio transceiver's evaluation and demo boards including the STEVAL SMARTAG1 (STMicroelectronics, Geneva, Switzerland) [72], RF430FRL152HEVM (Texas Instruments, Texas, USA) [71] and NHS3152TEMOADK (NXP, Eindhoven, Netherlands) [73]. This analysis showed that the STMicroelectronics's ST25DV16K NFC radio transceiver best met the requirements of the proposed application. Also, this NFC radio transceiver has the capability to harvest a maximum of 12.59 mW of power 5.5 A/m magnetic fields [8,74]. This level of energy transfer is sufficient to power up microcontroller as well as the temperature sensor and humidity sensor. Similarly, this NFC radio transceiver is also compliant with NFC type 5 specifications, and ISO/IEC15693 follows vicinity card standard [66]. Hence, this NFC radio transceiver can provide a communication range of 1 m with an ISO/IEC 15693 reader and up to 7 cm with NFC type 5-enabled smartphone [20].

An NFC sensor prototype was designed around the selected NFC radio transceiver, sensor and microcontroller to meet the defined requirements. Further, the proposed architecture of the NFC sensor was verified using components manufacturers' evaluation kits, and a first working prototype was developed using these evaluation boards such as X-NUCLEO-NFC04A1 (NFC radio transceiver) [75], NUCLEO-L031K6 (microcontroller) [76] and MIKROE-3331 Temp-Hum 9 click (temperature and humidity sensor) [77]. The NFC sensor architecture [80] and early stage results of the developed evaluation kit based prototype were reported by authors in [53] as a proof-of-concept.

S2: NFC sensor cost estimation

The cost estimation includes all the necessary components that are used to develop an NFC sensor such as, integrated circuits (IC), passive components, active components, printed circuits boards (PCB) manufacturing and electronics components assembly (including the shipping cost to Ireland from PCB manufacturer location in China). This cost estimate shows that the developed NFC sensor can be manufactured for approximately €4.91 per device in 10k quantities, as shown in Table S1. The cost estimate is based on quotations from an electronics components supplier, Digi-Key and a PCB manufacturer, PCBway [78,79]. For reference, this electronics components quotation has been generated online from Digi-Key Electronics Ireland website and for PCB manufacturing and components assembly from PCBWay website" on 2nd December 2020.

Table S1. Developed NFC sensor cost estimation at 10k quantities.

**Components Name**	**Component Description / Part Number**	**Cost per sensor (€)**
NFC radio transceiver	ST25DV16K-JFR6D3	0.54
Voltage regulator	STLQ015M18R	0.13
Microcontroller	STM32L031K6U6	1.16
Temperature and Humidity Sensor	SHTC3	0.99
PCB	PCB manufacturing and component assembly	1.03
Programming Connector	FFC FPC 12 POS connector	0.40
OR'ing Diode	Schottky diode array	0.04
Debug LED	Indication LED VF = 1.8 V, IF = 1 mA	0.13
MOSFET	N channel MOSFET 25V	0.08
Capacitors	X5R, 0402 package capacitors	0.19
Resistor	1/16W 0402, package resistor	0.06
Ferrite bead	Ferrite bead 600 Ohm	0.02
Connector	Conn header 2 POS 2.54 mm	0.14
	**Total Cost of Sensor (€)**	**4.91**

S3 Temperature measurement using the prototype NFC sensor

In order to determine the accuracy of the developed prototype sensor, the measured temperature values have also been compared with a calibrated Fluke 971 temperature and humidity meter. The testing was performed in the setup shown in Figure S1 using a cardboard box.

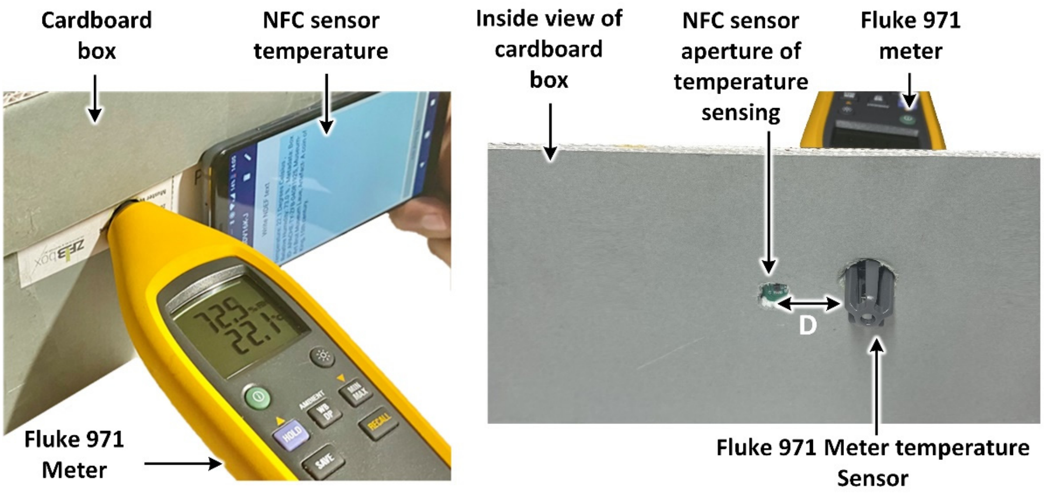

(a) (b)

Figure S1. NFC sensor temperature measurement setup **(a)** Outside view **(b)** Inside view.

As shown in Figure S1a,b sensing part of the fluke 971 is inserted inside the cardboard box. Likewise, the NFC sensor is integrated inside the cardboard box, and small aperture is created for temperature sensing, as described in the Section 2.5. The Fluke 971-meter and NFC temperature sensors were placed in close proximity within a distance D of 2.5 cm. For the measurement, lid of the cardboard box was closed, and the box was placed in different temperature conditions from 6.8 to 50.6 °C. The temperature of the NFC sensor was scanned from outside of the box, as shown in Figure S1b, simultaneously Fluke 971-meter temperature reading was recorded. Using this approach, 21 measurements have been taken, and the temperature value of the NFC sensor was compared with Fluke 971 meter. The measured temperature result is shown in Figure S2 with a minimum error of 0.2 °C and a maximum error of 0.6 °C being recorded. The above results show that the NFC sensor exhibited a mean error of ± 0.34 °C and a standard deviation of 0.361 °C in temperature. In Figure S2, the vertical bar symbols (in black) represent the magnitude of standard deviation, and the blue square symbols denote the mean error value.

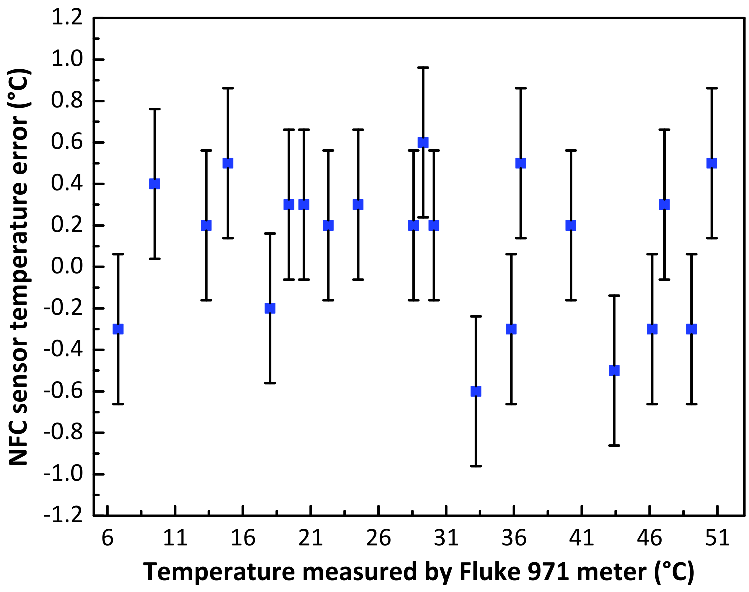

Figure S2. NFC sensor temperature measurement comparison with a calibrated Fluke 971 meter.

S4. Typical accuracy of relative humidity measurements

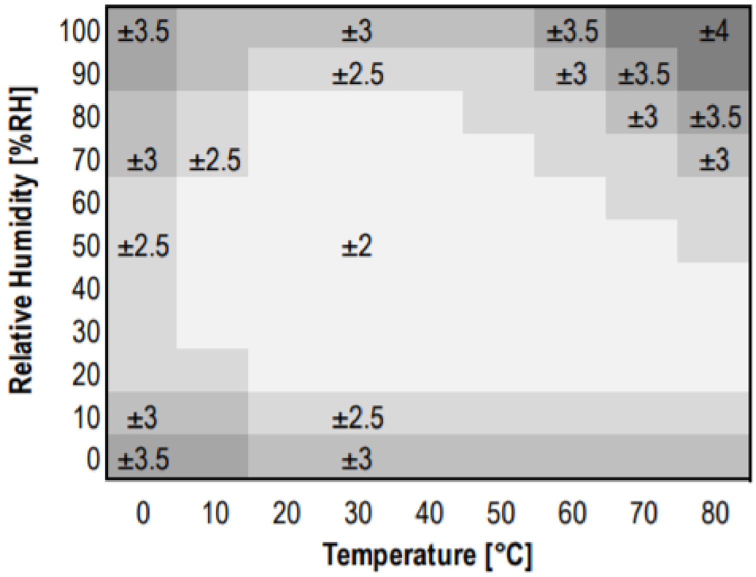

Figure S3. Typical accuracy of relative humidity measurements given in % RH for temperatures 0°C to 80°C [69].

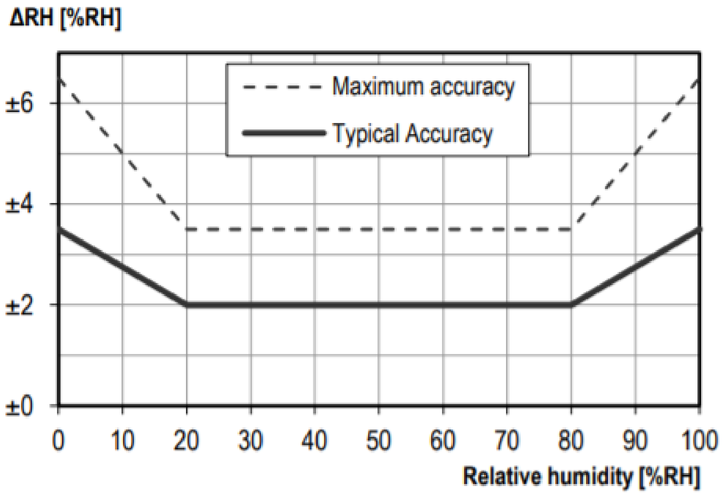

Figure S4. Typical and maximal tolerance for relative humidity at 25 °C [69].
